# Changes in the timing, length and heating degree days of the heating season in central heating zone of China

**DOI:** 10.1038/srep33384

**Published:** 2016-09-21

**Authors:** Xiangjin Shen, Binhui Liu

**Affiliations:** 1 College of Forestry, Northeast Forestry University, Harbin 150040, China; 2 Northeast Institute of Geography and Agroecology, Chinese Academy of Sciences, Changchun 130102, China

## Abstract

Climate change affects the demand for energy consumption, especially for heating and cooling buildings. Using daily mean temperature (Tmean) data, this study analyzed the spatiotemporal changes of the starting date for heating (HS), ending date for heating (HE), length (HL) and heating degree day (HDD) of the heating season in central heating zone of China. Over China’s central heating zone, regional average HS has become later by 0.97 day per decade and HE has become earlier by 1.49 days per decade during 1960–2011, resulting in a decline of HL (−2.47 days/decade). Regional averaged HDD decreased significantly by 63.22 °C/decade, which implies a decreasing energy demand for heating over the central heating zone of China. Spatially, there are generally larger energy-saving rate in the south, due to low average HDD during the heating season. Over China’s central heating zone, Tmean had a greater effect on HL in warm localities and a greater effect on HDD in cold localities. We project that the sensitivity of HL (HDD) to temperature change will increase (decrease) in a warmer climate. These opposite sensitivities should be considered when we want to predict the effects of climate change on heating energy consumption in China in the future.

Climate change has significant effects on many aspects of human society, including public health, water resources management, agriculture, and power generation^1^. The potential influence of climate change on energy demand for heating and cooling of buildings is especially important for developing countries^2^. In China, the largest developing country, the energy consumption of buildings reportedly accounts for 20–30% of the country’s total energy consumption^3^. Of that, up to 85% goes to heating in the north central heating zone and is mainly produced by burning coal, which can cause serious air pollution^4^. Unlike most other countries, China adopts artificial central heating system (citizens do not have control over heating their own places of residence) in the north to keep indoors warm in cold season. Precisely predicting the heating energy consumption in China is important for energy management and pollution control.

As a simple and well-known method for energy analysis, the degree-day method^5^,^6^ has been widely used for estimating energy requirements of buildings^7^,^8^. Many previous studies have investigated the impact of climate change on heating degree-days in China and other countries^1^,^9^,^10^,^11^,^12^,^13^,^14^,^15^,^16^,^17^,^18^,^19^. There are some studies that have found that heating degree days have significantly decreased in China during the past decades^17^,^20^,^21^,^22^,^23^. However, most of these studies only focused on the heating degree days throughout the year, and few have investigated the timing, length and heating degree days of heating season in China. Understanding the changes in both duration and quantity of heating energy consumption for warming buildings will provide important basic information for formulating energy policies in a changing climate. Therefore, an analysis of the influence of climate change on the intensity and duration of heating season is needed to understand heating energy consumption in China, especially in the north central heating zones.

In this study, using the daily mean temperature data from 437 stations in China (Fig. 1), we investigated the spatial and temporal changes of the starting date (HS), ending date (HE), length (HL), and heating degree-day (HDD) of heating season in the region of China that employs central heating system (namely, the central heating zones of China) during recent decades and investigated their relationships with air temperature. This study should provide valuable information for national energy management and planning in China.

## Results and Discussion

### Spatiotemporal Variation of Heating Variables in Central Heating Zone of China

Over the central heating zone of China, regional averaged start date for heating (HS) has become later by 0.97 day per decade and end date for heating (HE) has become earlier by 1.49 days per decade during 1960–2011, decreasing the length of the heating season (HL) by 2.47 days per decade. All the changes are statistically significant at the 99% confidence level and correspond with the general warming trend in China. During the study period, regional averaged heating degree days (HDD) significantly decreased at a rate of 63.22 °C/decade, implying a decreasing energy demand for heating throughout the central heating zone of China. This decreasing rate of regional HDD over the central heating zone of China is faster than that reported for the whole China (58.47 °C/decade) during 1961–2006 21. The different research regions selected in the research may account for this difference.

In terms of temporal change, we find large interannual variability for all the heating variables (Fig. 2). In general, both the timing and length of the heating season showed no significant change before the mid-1980. However, HS became later and HE came earlier after 1985, resulting in a rapid decline of HL (Fig. 2). For the HL, it decreased continually from 1985 to 2006 (with distinct reversal around 1992, corresponding with the cooling caused by the Pinatubo eruption in 1991), and reversing again after 2006. The temporal change of HL was most similar to that of HE, suggesting that HE may have a greater influence on HL than HS. Turning to the temporal variation in HDD, it fluctuated before the 1970 s, remained stable from 1970 to 1985, showed a rapid decrease from the 1985 to 1990, reached a stable level during 1990–2006, and then increased significantly after 2006 (Fig. 2). Although HDD values were calculated for the heating season, the temporal change of HDD was unlike that of HL, indicating that these changes are more complex than simply being directly related to temperature changes.

Figure 3 shows the spatial patterns of trends in HS, HE, HL and HDD for 238 stations in central heating zone of China from 1960 to 2011. Consistent with the regional trends, most stations reported later HS and earlier HE, thus producing a shorter HL. But the significant changes of HS and HE were only found in 61 and 116 of the 238 stations, respectively. Spatially, the greatest changes of HS were mainly observed in regions at high latitudes, such as Northwest and Northeast China, while the higher rates of HE change concentrated in the south of central heating zone (Fig. 3). As the magnitude of HE change is larger than that of HS, the spatial pattern of HL trends was similar to that of HE trends, i.e., the largest decreasing rates of HL were found in the south of the central heating zone of China. Consistent with the change in HL, there were remarkable negative trends of HDD at most stations, with only four stations showing no significant change (Fig. 3). However, the spatial patterning of HDD trend is unlike that of HL trend. During the study period, the largest decreases of HDD were found in northeast China, central region of Inner Mongolia, Qinghai Province and northern regions of Xinjiang (Fig. 3). The different spatial patterns of trends in HL and HDD indicate that these variables respond differently to a changing climate.

### Relationships between Heating Variables and Mean Temperature

The results of correlation analysis showed that the relationship of annual HL with HE was larger than that with HS (Table 1). This confirms that the change of end date has a greater impact on the length of the heating season in China. Seasonally, the largest correlation between mean air temperatures and heating variables were found in autumn for HS (r = 0.82), spring for HE (r = −0.84), and heating year for HL (r = −0.81) and HDD (r = −0.95), respectively. These results are reasonable because the start and end data for heating mainly occur in autumn and spring respectively; while both HL and HDD reflect the whole heating season.

As is well documented, seasonal mean temperatures have risen in the central heating zone of China, especially since 1985, with the greatest warming in winter^24^. We found that there are obvious spatiotemporal patterns of seasonal mean surface air temperature changes in northern China, with a larger warming in winter (Fig. 4). These results are consistent with many previous studies^24^,^25^. In this study, the temporal changes of HS, HE and HL were similar to that of Tmean in autumn, spring and heating year, respectively (Fig. 4). No significant changes of seasonal Tmean before the mid-1980 s and rapid increases of seasonal Tmean after 1985 account for the changes of corresponding heating variables during these two periods. Although the correlation between HDD and heating year Tmean was the largest, the temporal change of HDD was more like that of winter Tmean especially during 1990–2006 when both HDD and winter Tmean reached a stable level (Fig. 4).

Considering the rapid warming of Tmean after 1985, we compared the correlations among variables during two periods, 1960–1984 and 1985–2011. There were small but meaningful differences between the correlations among variables during two different periods (Table 1). In the earlier period, the significant correlation between seasonal Tmean and annual HL was only found for spring Tmean. But in the later period, there were comparable and significant relationships of annual HL with autumn, winter, and spring Tmean. In addition, the correlation between annual HDD and winter Tmean was greater in the later period. Although we cannot conclude that these correlations are significantly different considering relatively small sample sizes (25–26 years), the correlations using detrended data show not much change between the two periods (Table 2). It seems that, as the climate warms, temperature changes in autumn and winter begin to have some effects on HL, and the effects of winter temperature on heating energy demand in China become much larger.

By plotting the trends in mean temperatures and heating variables on maps, we found that the spatial characteristics of the autumn Tmean trend (Fig. 5a) generally resemble the pattern for HS across China’s central heating zone (Fig. 3a), confirming the effects of autumn temperature changes on HS. But in Inner Mongolia, the high warming rates of autumn Tmean did not see significant change of HS (Figs 3a and 5a). As with the trend of spring Tmean, the spatial pattern of it differs from that of the HE, with the higher rates of spring Tmean increase concentrating in Inner Mongolia, and west regions of Heilongjiang Province and Jilin Province (Fig. 5b). The spatial differences of trends in spring Tmean and autumn Tmean partly account for the spatial heterogeneity of HE and HS trends. In addition, the existed spatial differences of trends in HE and spring Tmean (HS and autumn Tmean) indicate that the sensitivity of HE (HS) to temperature change may exist some spatial differences. Similarly, the spatial patterns of change in heating year Tmean is unlike that of both HL and HDD (Figs 3c,d and 5d). These above differences indicate that the relationship between heating variable and average temperature may have spatial difference. Although with some differences, we found that the spatial patterns of trends in HDD was more like that of winter Tmean (Figs 3d and 5c), suggesting that the change of winter Tmean plays the most important role in HDD changes.

To further discuss the relationships between heating variables and mean temperatures, we investigated the sensitivity of heating variables to temperature changes. For each station, a scatter plot of the 1960–2011 values of heating variables versus seasonal Tmean produced a linear distribution. Following previous studies^26^,^27^, we computed the slope of each regression line (expressed as change in heating variables with a 1 °C rise in average temperature) as a climate sensitivity response parameter to indicate the sensitivity of heating variables to temperature changes in this study. For the timing of heating season, larger sensitivity of HS (HE) to autumn Tmean (spring Tmean) were mainly found in the south of central heating zone (Fig. 6). As a result, these regions see larger sensitivity of HL to heating year Tmean. By contrast, more sensitive responses of HDD to heating year Tmean occurred in northeast China, central region of Inner Mongolia, Qinghai Province and northern regions of Xinjiang (Fig. 6). These spatial patterns of sensitivities account for the spatial differences of trends in heating variables and seasonal Tmean.

Among different stations, different sensitivities of HL and HDD to heating year Tmean may be related to geographical conditions of these localities. As can be seen from the maps of HL and HDD slopes against heating year Tmean (Fig. 6c,d), the sensitivity of HL to heating year Tmean was larger in southern, warmer localities, but larger sensitivity of HDD to heating year Tmean occurred in northern, colder localities. It is an interesting finding because it implies opposite responses of HL and HDD to temperature change. That is to say, the impacts of temperature change on HL may be more obvious in southern, warmer localities, but the effects on HDD may be more obvious in northern, colder localities. The reason for this difference may be related to different temperature conditions of these localities. In the south, winter temperatures are closer to the threshold temperature, so HL becomes more sensitive to changes in mean temperature. But in the north where is always cold and below the threshold, the changes of accumulated HDD caused by the temperature variation are much stronger due to longer heating period. Therefore, if the whole central heating zone were to see the same degree of warming, the HL in the south would become much shorter than that in the north, but HDD decrease more drastically in the cold. Under the background of climate warming, we can predict that the sensitivity of HL to temperature change will increase, whereas that of HDD will decrease as the climate warms. These different responses of HL and HDD to temperature change should be considered when we want to predict heating energy consumption in China in a changing climate.

### Impact of Temperature Change on Potential Heating Energy Consumption in China

Based on the equation (1), we calculated the heating energy-saving rate (by comparing the HDD difference during two periods of 1985–2011 and 1960–1984) in China caused by temperature increase. The results showed that the heating energy consumption decreased about 13.42% over the central heating zone of China due to climate warming after the mid-1980 s. Spatially, larger energy-saving rate concentrated in south warming localities (Fig. 7a), and the energy-saving rates for the south provinces and autonomous regions were generally larger than that for the north provinces and autonomous regions (Fig. 7b). For example, the heating energy-saving rate for Hebei province was 19.77%, which was obviously larger than that for three northeast provinces of Heilogjiang (9.28%), Jilin (10.58%) and Liaoning (11.91%) (Fig. 7b). Similarly, the heating energy-saving rate for Shanxi (15.94%), Ningxia (16.65%) and Tibet (14.09%) were larger than that for the north provinces and autonomous regions. The reason for larger heating energy-saving rate in warming localities is mainly due to relatively low HDD during the heating season (Table 3).

We recognize that anthropogenic influence especially the urbanization effect on the changes of heating energy consumption should not be ignored though it is not the focus of the current study. Urbanization has proceeded at an unprecedented rate in China since the mid 1980 s^28^, urban construction and population growth would tend to increase the aggregate demand for heating though this can be mitigated with increased energy efficiency^29^. Some previous studies demonstrated that urbanization has made a big contribution to the observed warming^30^,^31^,^32^. A new study found that urban warming influences account for about a third of the observed warming in China over the period 1961–2013^33^. Considering the significant urbanization effect in the long-term temperature data series in China, urbanization may bring some observational bias in the surface air temperature data series and thus has some effects on the trend estimates of the indicators of heating. Therefore, the current study may overestimate the effect of climate change on potential energy consumption in the country. However, further research is still needed to investigate how much effect the urbanization has had on the changes of heating energy consumption in China.

## Conclusions

Based on daily average temperature records of China, this study analyzed the spatiotemporal changes in HS, HE, HL and HDD in central heating zones of China during 1960–2011. Over the central heating zone of China, regional averaged HS has become later and HE has become earlier since the mid-1980 s, decreasing the HL by 2.47 days per decade during 1960–2011. The decrease of HL was mainly determined by earlier HE due to increase of spring Tmean.

During 1960–2011, regional averaged HDD decreased at a rate of −63.22 °C/decade, implying a decreasing energy demand for heating throughout the central heating zone of China. Due to climate warming after the mid-1980 s, the heating energy consumption (relative changes of HDD between 1985–2011 and 1960–1984) decreased about 13.42% over the central heating zone of China. Spatially, there are generally larger energy-saving rate in southern, warmer localities, because of lower average HDD during the heating season.

With climate warming, winter temperature changes could have larger effects on heating energy demand in China. The increase of heating year Tmean will result in obvious decline of both HL and HDD. But the HL and HDD respond to the changes of heating year Tmean in an opposite way. The sensitivity of HL to heating year Tmean was larger in southern, warmer localities, but larger sensitivity of HDD to heating year Tmean occurred in northern, colder localities. Therefore, we can predict that the sensitivity of HL to temperature change will increase as the climate warms, while the sensitivity of HDD will decrease. These different responses of HL and HDD to temperature change should be considered when we want to predict heating energy consumption in China in a changing climate.

## Methods

### Materials

**Figure 1:**
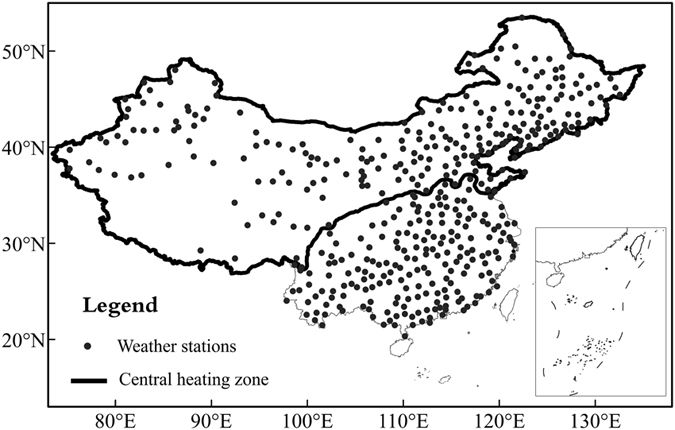
The distributions of the 437 weather stations and central heating zone of China. The software ArcGIS 10.0 was used to create the map.

**Figure 2:**
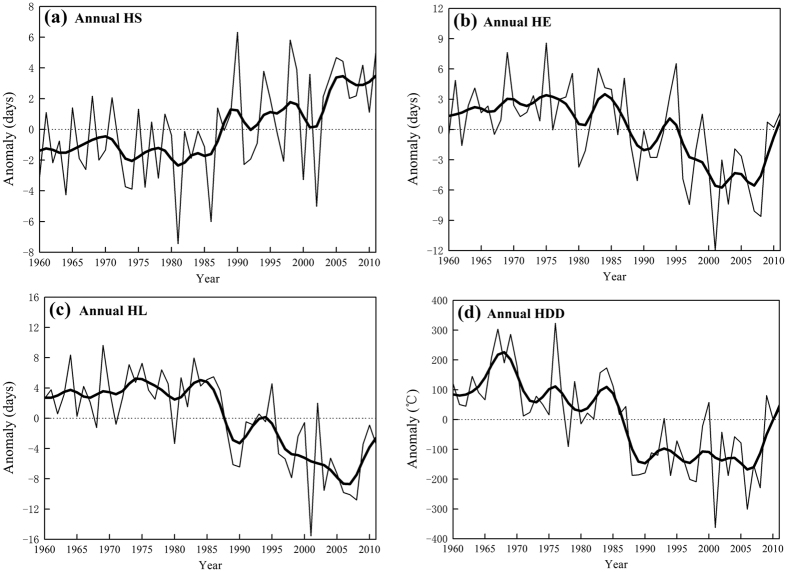
Anomaly series (vs normal 1960–2011 values) of regional average values of annual HS, HE, HL, and HDD for central heating zone of China. The heavy line is the result of smoothing with a 9-year binomial filter with reflected ends.

**Figure 3:**
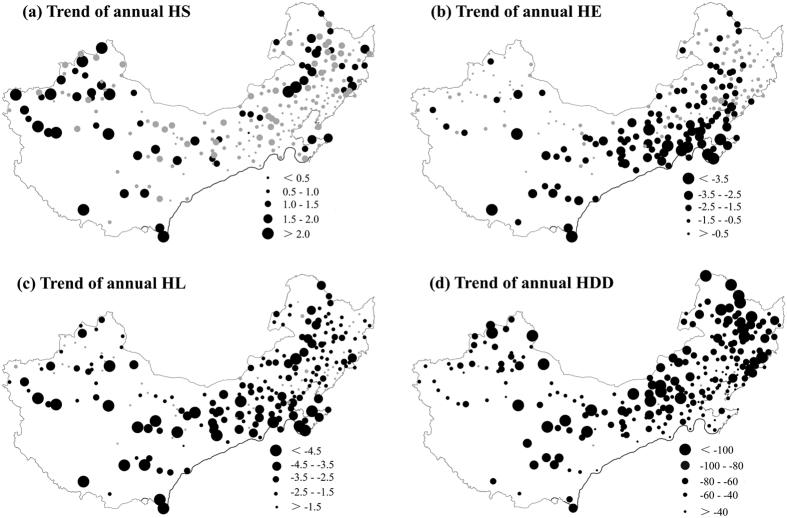
The spatial patterns of decadal trends in HS (days/decade), HE (days/decade), HL (days/decade) and HDD (°C/decade) for 238 stations in central heating zone of China during 1960–2011. The light-colored circles indicate no significant change. The maps were generated using ArcGIS 10.0 software.

**Figure 4:**
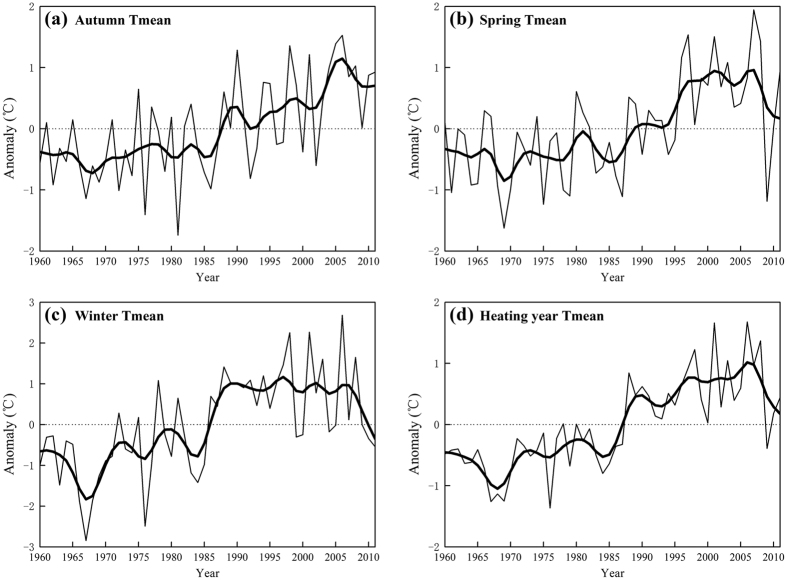
Same with Fig. 2 but for seasonal temperatures.

**Figure 5:**
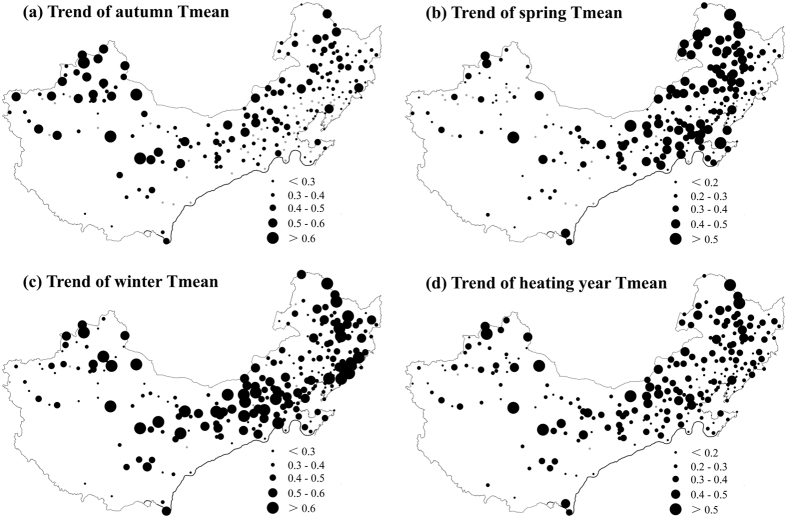
Same with Fig. 3 but for trends in temperatures (°C/decade). The maps were generated using ArcGIS 10.0 software.

**Figure 6:**
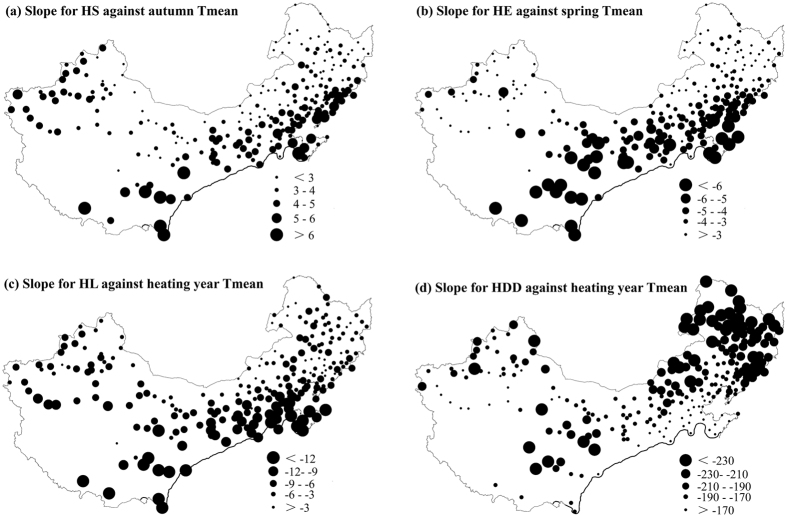
Slope of heating variables against corresponding Tmean for each station in the central heating zone of China. The maps were generated using ArcGIS 10.0 software.

**Figure 7:**
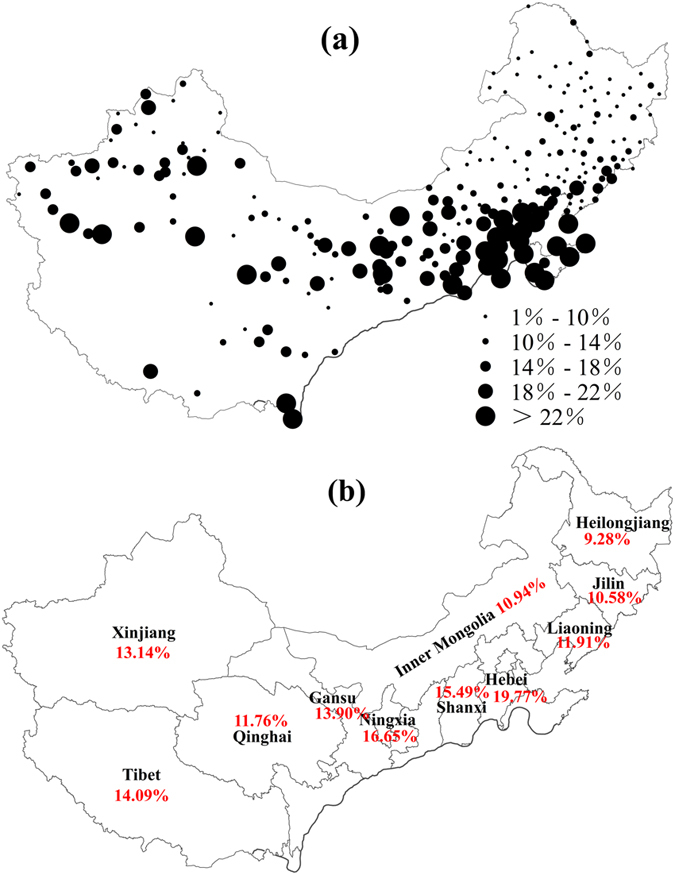
The energy-saving rate of heating energy consumption in central heating zone of China due to climate warming, expressed by the relative changes of HDD between the period of 1985–2011 and 1960–1984: (**a**) each station, (**b**) all the provinces and autonomous regions. The maps were generated using ArcGIS 10.0 software.

In this study, daily mean temperature (Tmean) records from 437 stations in China during 1960–2012 were selected for analysis (Fig. 1). Following the procedure described in our previous paper^34^, we conducted vigorous data assurance assessment to assure the integrality and homogenization of the data. It is inevitable that long-term monitoring datasets include some missing data; here, missing data accounted for 0.39% of the total records during 1960 to 2011, and we adopted the method used by Liu *et al*.^24^ to fill these data gaps.

### Definition of heating indices

According to Design Code for Heating Ventilation and Air Conditioning of Civil Buildings^35^, central heating zone of China is defined as regions where the cumulative annual length of heating season is no less than 90 days. The length of heating season for each year is determined by the cumulated days when daily average outdoor temperature is steadily less than or equal to 5 °C^35^, namely, all the 5-day running mean temperatures are less than or equal to 5 °C during the heating season of this year. For each year at each station, a 5-day running mean method is adopted to determine the HS and HE, following the method for calculations of the start and end dates used by Liu *et al*.^36^. First, we identified the longest period that the 5-day running mean temperatures are steadily less than or equal to 5 °C, noting Xb and Xe, the beginning and ending dates of this period. HS is then determined to be the first day between dates Xb − 2 and Xb + 2 that the Tmean drops less than or equal to 5 °C; HE was calculated as the last day between dates Xe − 2 and Xe + 2 that the Tmean remains less than or equal to 5 °C^36^. HL is then calculated as the number of days from HS to HE, inclusive.

Generally, HDD are calculated at a base temperature in the vicinity of 16–20 °C for most countries where the citizens control over heating their own places of residence^11^,^13^,^15^. However, these base temperatures for calculating HDD are not suitable for China^37^,^38^,^39^, where the government controls the central heating system. Considering the average housing insulation properties in north China, without artificial heating condition, the indoor average temperature is around 10–12 °C when the outdoor average temperature is 5 °C^40^. According to the national standard on the climate conditions of heating demand^41^, the base temperature of 5 °C is adopted for calculating HDD in China^37^,^38^,^39^,^40^. By contrast with developed countries, China’s base temperature for heating is obviously low, but it is predicted to increase with the improvement of living standards in China^42^. For this paper, we define the HDD in each year as the sum of the difference between base temperature and daily average temperature when daily average temperature is below the base temperature during the heating season. Considering the annual pattern of heating season, we calculated the heating variables for each year based on the daily temperature data from July 1st to June 30th. To better understand the relationship of air temperature with different heating variables, we defined a “heating year” which can be divided into autumn (September to November), a winter transition period (December to February), and spring (March to May).

It is reported that the value of accumulated HDD during the heating season is directly proportional to energy consumption for heating buildings^37^. If the energy consumption with respect to per unit of HDD are basically the same for a particular region, the percentage of HDD decrease can be expressed as energy-saving rate of heating energy consumption in this region^43^,^44^. In a particular region, the heating energy-saving rate (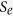) due to climate warming can be calculated as:
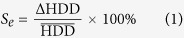
where 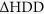 is the HDD difference during two periods before and after climate warming, and 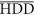 is the average HDD during the whole period.

### Data processing

To obtain the spatial patterns of cumulative HL during the study period, we used an inverse distance weighted interpolation method. Based on the counter map, we determined the central heating zone of China where the cumulative HL 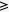 90 days^35^, and selected it as the study area (Fig. 1). The central heating zone stretches across China’s northern tier from the Tibetan Plateau to the Shandong peninsula, encompassing all or part of 17 provinces, municipalities and autonomous regions, and accounting for about 70 percent of the country’s territory. Regional average values of heating variables in the central) heating zones of China were computed by the Thiessen polygon method^45^. To smooth out the year-to-year variations in a time series, we applied a nine-point binomial filter to analyze temporal variation of variables. The anomalies of temperature variables were calculated based on the whole study period of 1960–2011. We estimated trends using the Mann-Kendall (MK) test and simple linear regression^46^,^47^.

## Additional Information

**How to cite this article**: Shen, X. *et al*. Changes in the timing, length and heating degree days of the heating season in central heating zone of China. *Sci. Rep.* **6**, 33384; doi: 10.1038/srep33384 (2016).

## Figures and Tables

**Table 1:** Correlation among the heating and temperature variables, with the largest correlation coefficients showed in bold.

	HS	HE	HL	HDD	Autumn Tmean	Winter Tmean	Spring Tmean	Heating year Tmean
1960–2011								
HS	1	−0.11	−0.64**	−0.44**	0.82**	0.30*	0.15	0.51**
HE	−0.11	1	0.83**	0.65**	−0.25	−0.52**	−0.84**	−0.68**
HL	−0.64**	0.83**	1	0.74**	−0.65**	−0.57**	−0.73**	−0.81**
HDD	−0.44**	0.65**	0.74**	1	−0.66**	−0.92**	−0.58**	−0.95**
1960–1984								
HS	1	0.22	−0.48**	−0.15	0.64**	−0.08	−0.16	0.19
HE	0.22	1	0.75**	0.36	0.22	−0.16	−0.79**	−0.34
HL	−0.48*	0.75**	1	0.43*	−0.24	−0.09	−0.59**	−0.43
HDD	−0.15	0.36	0.43*	1	−0.49**	−0.82**	−0.28	−0.96**
1985–2011								
HS	1	0.19	−0.48	−0.23	0.85**	0.09	−0.21	0.32
HE	0.19	1	0.77**	0.60**	−0.01	−0.52*	−0.75**	−0.67**
HL	−0.48**	0.77**	1	0.67**	−0.53*	−0.52*	−0.56*	−0.80**
HDD	−0.23	0.60**	0.67**	1	−0.42	−0.88**	−0.41	−0.90**

*P < 0.05; **P *<* 0.01.

**Table 2:** Correlation among the detrended heating and temperature variables during two different periods, with the largest correlation coefficients showed in bold.

	HS	HE	HL	HDD	Autumn Tmean	Winter Tmean	Spring Tmean	Heating year Tmean
1960–1984								
HS	1	0.31	−0.46*	−0.10	0.65**	−0.08	−0.29	0.07
HE	0.31	1	0.71**	0.14	0.31	0.11	−0.81**	−0.16
HL	−0.46*	0.71**	1	0.20	−0.11	0.17	−0.54**	−0.21
HDD	−0.10	0.14	0.41*	1	−0.42*	−0.86**	−0.11	−0.92**
1985–2011								
HS	1	0.23	−0.44*	−0.30	0.81**	0.16	−0.21	0.32
HE	0.23	1	0.77**	0.69**	0.00	−0.11	−0.73**	−0.23
HL	−0.44*	0.77**	1	0.83**	−0.33	−0.17	−0.54**	−0.19
HDD	−0.30	0.69**	0.83**	1	−0.47**	−0.85**	−0.24	−0.92**

*P *<* 0.05; **P *<* 0.01.

**Table 3:** Changes of HDD between the period of 1985–2011 and 1960–1984 for the main provinces and autonomous regions in the central heating zone of China.

	Change of HDD (°C)	Average HDD (°C)	Change rate of HDD (%)
Heilongjiang	−270.95	2936.68	9.28
Jilin	−231.00	2209.02	10.58
Liaoning	−160.21	1410.84	11.91
Hebei	−182.97	1085.26	19.77
Inner Mongolia	−247.02	2454.74	10.94
Shanxi	−157.64	1203.73	15.49
Qinghai	−203.54	1903.46	11.76
Gansu	−189.04	1376.49	13.90
Ningxia	−200.73	1220.36	16.65
Xinjiang	−184.31	1538.05	13.14
Tibet	−160.47	1189.73	14.09
